# Scientific Items

**Published:** 1883-03-20

**Authors:** 


### SCIENTIFIC ITEMS.

**Some Large Lenses.**—The thirty-inch objective for the great telescope of the Russian Observatory at Pulkova was lately tested at the entertainment of the grinders, the Clarks, of Cambridgeport, Massachusetts, and found to be fairly perfect. The flaw discovered before the grinding, due to imperfect cooling, has no effect on the definition, but lessens slightly the amount of light transmitted. The flaw is too slight to injure materially the efficiency of the lens, yet another block of glass, of the same size, has been ordered to be placed at the disposal of Professor Struve. For testing, the lens is mounted in a temporary telescope, forty-five feet long, and weighing, with its fittings, about seven tons. The lens weighs 450 pounds, will cost, when finished, $60,000, and will be, for a little while, the largest in the world.

The largest object-glass in use is the 26-inch lens at Washington, with a focal length of 33 feet. Its light-gathering power is 16,000 times that of the unaided eye.

The Pulkova glass will soon be excelled by that of the Lick telescope, the disk of glass for which is now in the establishment of the Clarks. It is 38 inches in diameter and 2 inches thick. When ground and polished it will be reduced to 36 inches. The glass is optically perfect. It was cast at Paris, France, where the Pulkova glass was, and weighs a little over 374 pounds. The casting occupied four days, and the cooling thirty days.—*Scientific American.*

**The average** birth rate per annum in France, for the period between 1872 and 1880, has been calculated to be one birth for 37 inhabitants, which is by far the lowest birth rate in Europe. For the different countries the birth rate is as follows: Russia, one birth for 20 inhabitants; Germany, one birth for 25; Austro-Hungary, one birth for 26; England, one birth for 27; Italy, one birth for 27; Spain, one birth for 28; France, one birth for 37. If the yearly number of births for any thousand inhabitants be calculated, we have precisely the same result. We have in France, 26 births per 1,000; Belgium, 32; England, 35; Austria, 38; Prussia, 38; Russia, 50, and the United States, 55.—*Journal of Health.*

**Lightning Rods.**—Two things are to be considered in conveying the electricity from the air to the earth: the collecting and distributing of the fluid as it is generated. The collecting is best accomplished by means of metallic points above the object to be protected. These points should be in metallic connection, by means of rods, or continuous metallic parts, that may enter into the construction of the structure, with the earth.

As the rods have an enormous capacity for conveying electricity, compared to building materials, except the metals, there must be abundant facilities for disposing of the current, as well as collecting it. Simply terminating a rod in the ground, or even in water, is not sufficient, for they are such poor conductors that much greater surface must be presented to the metallic conductor, in order to carry off an unappreciable amount of electricity. Since the conductive power is in proportion to the area of the cross-section of the conductor, it requires many square feet of earth or water to distribute what may readily pass through a metallic rod only a small portion of an inch in sectional area.

There is, then, no better practical method for securing a good grounding for a conductor than connecting it with a water or gas main, which, having very extensive surface contact with damp or wet earth, serves as an excellent distributor. A conductor passing to the water should terminate in a large metallic plate, so as to facilitate the passage of the current from the rod to the water.—*Mechanical News.*

**A Curious Experiment.**—The ease with which persons fall under hallucinations of special sense is illustrated by M. Yung, in a recent communication to the Helvetic Society of Sciences. The operator places eight cards on a table, in positions corresponding to forehead, eyes, ears, nose, mouth and chin; he pretends to "magnetize" them and also some person in the company, and then goes out, while the magnetized person is required to touch any one card. The operator, having returned, notes the action of a confederate, who scratches a part of his head corresponding to the card touched. Then he commences an innocent comedy, passing his hand carefully over the cards, and on reaching the touched card seeming to experience a strong shock. The observers are surprised, of course. One of them is then asked to go out and repeat the experiment. It is assumed that a certain card has been touched. Passing his hand over the cards, he indicates, in nine cases out of ten, a particular card as giving him a shock; and if the company be instructed to support his idea of that being the "correct card," he is confirmed in his illusion, which may be successfully repeated. Of 85 persons tried, M. Yung found only 9 who refused to indicate a card, not having experienced any sensation; 53 said they had exactly the sensation announced, and 23 described some different sensation.—*Journal of Chemistry.*

**The Climate of Palestine.**—From its peculiar formation the country possesses much variety of climate. That of the hill country has been compared with the climate of Italy, while that of the Jordan valley is decidedly tropical. The rainy season usually commences towards the end of October, and lasts till March, after which the air clears, and for months the bright blue sky is unbroken by a single cloud. The annual rainfall is small, the average of seven years, during which observations have been taken, being only nineteen inches and a half.—*Ibid.*

